# Strong genotype‐by‐genotype interactions between aphid‐defensive symbionts and parasitoids persist across different biotic environments

**DOI:** 10.1111/jeb.13953

**Published:** 2021-11-02

**Authors:** Elena Gimmi, Christoph Vorburger

**Affiliations:** ^1^ Department of Aquatic Ecology Eawag, Swiss Federal Institute of Aquatic Science and Technology Dübendorf Switzerland; ^2^ Department of Environmental Systems Science D‐USYS ETH Zürich Switzerland

**Keywords:** aphids, defensive symbiosis, genotype‐by‐genotype interactions, host–parasite coevolution, parasitoids, resistance

## Abstract

The dynamics of coevolution between hosts and parasites are influenced by their genetic interactions. Highly specific interactions, where the outcome of an infection depends on the precise combination of host and parasite genotypes (G × G interactions), have the potential to maintain genetic variation by inducing negative frequency‐dependent selection. The importance of this effect also rests on whether such interactions are consistent across different environments or modified by environmental variation (G × G × E interaction). In the black bean aphid, *Aphis fabae*, resistance to its parasitoid *Lysiphlebus fabarum* is largely determined by the possession of a heritable bacterial endosymbiont, *Hamiltonella defensa*, with strong G × G interactions between *H*. *defensa* and *L*. *fabarum*. A key environmental factor in this system is the host plant on which the aphid feeds. Here, we exposed genetically identical aphids harbouring three different strains of *H*. *defensa* to three asexual genotypes of *L*. *fabarum* and measured parasitism success on three common host plants of *A*. *fabae*, namely *Vicia faba*, *Chenopodium album* and *Beta vulgaris*. As expected, we observed the pervasive G × G interaction between *H*. *defensa* and *L*. *fabarum*, but despite strong main effects of the host plants on average rates of parasitism, this interaction was not altered significantly by the host plant environment (no G × G × E interaction). The symbiont‐conferred specificity of resistance is thus likely to mediate the coevolution of *A*. *fabae* and *L*. *fabarum*, even when played out across diverse host plants of the aphid.

## Graphical abstract

Environmental variation may affect the outcome of host‐parasite interactions. We show that aphid host plants—here from left to right *Beta vulgaris*, *Chenopodium album* and *Vicia faba*—quantitatively influence parasitism success of a parasitoid wasp on symbiont‐protected aphids, however without altering the strong G × G interactions occurring between parasitoids and aphid‐defensive symbionts.

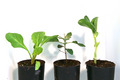

## 1 INTRODUCTION

Whether the encounter between a host and a parasite leads to infection or not is often determined by the distinct combination of their respective genotypes. In classical infection matrix experiments, where multiple host and parasite genotypes are exposed to each other, such genotype specificity manifests itself in genotype‐by‐genotype (G × G) interactions on the probability of infection (e.g. Carius et al., 2001; Salvaudon et al., 2007; Schulenburg & Ewbank, 2004). G × G interactions may contribute to the maintenance of genetic diversity within species through negative frequency‐dependent selection: a frequent host or parasite genotype exerts selection on its antagonist to counter‐adapt and is soon going to be at a disadvantage compared to rare genotypes. Many rare genotypes are therefore favoured over few frequent ones in the long term (Clarke, 1976; Judson, 1995). G × G interactions may also contribute to the evolutionary maintenance of sexual reproduction and recombination, which promote diversity by combining existing genes and producing new, rare offspring genotypes (The Red Queen Hypothesis: Bell, 1982; Hamilton, 1980; Hamilton et al., 1990; Jaenike, 1978).

The potentially far‐reaching influence of G × G interactions on host–parasite coevolution also hinges on their sensitivity to environmental variation. With the environment varying across the geographic distribution of interacting species, also the conditions for successful host infection or defence can change, potentially altering the strength and direction of selection acting on host and parasite (Nuismer et al., 2000; Tétard‐Jones et al., 2007; Thompson, 2005; Wolinska & King, 2009). Different genotypes of a species may respond unequally to changing environmental conditions, resulting in genotype‐by‐environment (G × E) interactions that influence the outcome of host–parasite encounters. Moreover, the strength and specificity of G × G interactions may be dependent on the environment, which is referred to as a genotype‐by‐genotype‐by‐environment interaction (G × G × E). Such three‐way interactions may contribute to the maintenance of genetic diversity at the species level as well, and they make the outcome of genotype‐specific selection of hosts and parasites less predictable (Mostowy & Engelstädter, 2011; Wolinska & King, 2009). Experimental evidence for G × G interactions that are sensitive to environmental variation, that is for G × G × E interactions, is not abundant but exists for diverse systems. Examples include G × G × E interactions with the rhizosphere environment influencing the outcome of specific interactions between plants and herbivore genotypes (Tétard‐Jones et al., 2007), or the food environment influencing the G × G interactions that determine parasite infection in bumblebees (Sadd, 2011). Similarly, Bryner and Rigling (2011) found that temperature interacts with the genotypes of tree pathogenic fungi and their hyperparasitic viruses when predicting fungal virulence, and a three‐way interaction between temperature, host genotype and parasite genotype may determine the transmission potential of viral diseases by mosquito vectors (Zouache et al., 2014). Apart from experimental evidence, modelling approaches support the potential influence of environmental changes on host–parasite coevolution through three‐way interactions (Mostowy & Engelstädter, 2011). Taken together, these references underline the importance of incorporating environmental variability into classical G × G interaction studies, prior to generalizing conclusions to more complex natural systems.

Aphids have become popular model organisms for studying host–parasite interactions, not least because of the fascinating way by which certain species resist natural enemies: they carry defensive symbionts protecting them, for example, against pathogenic fungi (Lukasik et al., 2013; Scarborough et al., 2005) or parasitoid wasps (Asplen et al., 2014; Oliver et al., 2003; Vorburger et al., 2009). The best‐studied example for the latter is the gammaproteobacterium *Hamiltonella defensa* (Moran, Russell, et al., 2005; Oliver et al., 2003), a maternally transmitted endosymbiont. There are different strains of the endosymbiont *H*.* defensa* which may confer different levels of resistance against parasitoid wasps, depending on the species or also the genotype of the attacking parasitoids (Asplen et al., 2014; Cayetano & Vorburger, 2015). Because maternal transmission of *H*. *defensa* is very reliable (Darby & Douglas, 2003; Peccoud et al., 2014; Vorburger et al., 2017), *H*. *defensa* may be regarded as a form of a selectable resistance trait of its aphid host, with different strains acting as different genotypes determining the characteristics of this trait (Jaenike, 2012). Since *H*.* defensa*‐conferred resistance tends to be much stronger than the basal resistance of the aphid (Oliver et al., 2005; Vorburger et al., 2009), coevolution between aphid hosts and parasitoids is likely mediated by defensive symbionts (Vorburger & Perlman, 2018).

The present study is concerned with the interaction between the black bean aphid, *Aphis fabae* (Hemiptera: Aphididae), and its main parasitoid *Lysiphlebus fabarum* (Hymenoptera: Braconidae, Aphidiinae). Different experiments have shown that the rate of successful parasitism in this system is determined by strong G × G interactions between *L*.* fabarum* and the aphids’ symbiont *H*.* defensa* (Rouchet & Vorburger, 2012; Schmid et al., 2012). This suggests a decisive role of *H*. *defensa* in governing natural coevolutionary dynamics between *A*. *fabae* and *L*. *fabarum* (Kwiatkowski et al., 2012). However, many of the experiments yielding the current knowledge were justifiably done under constant ambient conditions in the laboratory, which leaves open the question of how consistent—and thus relevant—such G × G interactions may be in heterogeneous natural environments. The only environmental variable that has been explicitly manipulated in this system is temperature: on the one hand, Cayetano and Vorburger (2013b) showed that G × G interactions between *L*.* fabarum* and *H*.* defensa* remain qualitatively the same at different ambient temperatures, even though the level of resistance conferred by *H*.* defensa* drops with increasing temperature, as also seen in pea aphids (Bensadia et al., 2006; Doremus et al., 2018). On the other hand, the same authors found that heat shocks experienced by the aphids could affect G × G interactions, albeit only at very high temperatures that are rarely experienced in nature (Cayetano & Vorburger, 2013a). We aimed to complement these studies by manipulating a different, yet crucially important environmental variable of the same host–endosymbiont–parasitoid system, namely the aphid's host plant.

Plants are both the food source and habitat of an aphid, and their availability and quality are an important determinant of seasonal and spatial environmental variation. There is ample evidence for plants having a direct influence on the host–parasite ecology of their insect inhabitants. For instance, Sochard et al. (2019) recently found that costs imposed on pea aphids (*Acyrthosiphon pisum*) by both parasitoids and endosymbionts depend on the aphid's host plant. And while Sochard et al. (2019) did not find any evidence for an influence of the host plant on parasitism rates, Goldson and Tomasetto (2016) found that parasitism rates in a weevil species are different depending on the grass species on which the weevils feed. Correlations between host plant, resistance to parasitoids and natural *H*. *defensa* infections of pea aphids led McLean et al. (2011) to suggest that selection pressure by parasitoids varies across different host plants. As shown for the same aphid species, also predation rates can be influenced by the host plant (Aquilino et al., 2005). Generally, host plant choice may affect the fitness of herbivorous insects and consequently the fitness of their predators or parasitoids (Pan et al., 2020).

While the influence of the host plant on insect interactions is striking in many systems, actual studies of G × G × E interactions with host plant as the environmental variable are rare. Investigating such interactions requires a study system where specific genotype combinations can be replicated, as is the case for the *A*.* fabae*/*H*.* defensa*/*L*.* fabarum* system. *A*. *fabae* can reproduce clonally, and its infection with *H*. *defensa* can readily be manipulated by microinjection (Oliver et al., 2003; Sochard et al., 2020), while the occurrence of asexual reproduction in *L*. *fabarum* (thelytoky, see Sandrock et al. (2011)) enables the use of distinct, genetically homogeneous lines also for the parasitoid. Environmental variability due to host plants is of high relevance for the multivoltine *Aphis fabae*, where opportunistic switches between crops and weeds from one aphid generation to the other are important to allow continuous feeding and reproduction over a whole growing season. We thus investigated the influence of three different, common host plants of *A*.* fabae* on G × G interactions between the aphid‐defensive symbiont *H*. *defensa* and the parasitoid *L*. *fabarum*, using a full factorial design. The host plant had a strong effect on overall parasitism rates and thus on wasp reproductive success, and it also affected aphid fitness independent of parasitism rates. The host plant did, however, not alter the strong G × G specificity between *L*. *fabarum* and *H*. *defensa*. Hence, our results support earlier studies, suggesting that in our model system, coevolution between aphids and parasitoids is largely symbiont‐mediated and governed by genotype‐specific interactions, which remain remarkably stable across different environments.

## 2 MATERIALS AND METHODS

### 2.1 Organisms

As host plants, we used broad bean (*Vicia faba*, var. Fuego, UFA Samen, Winterthur, Switzerland), the common goosefoot (*Chenopodium album*) and green chard (*Beta vulgaris*, var. Grüner Schnitt, Samen Mauser AG, Winterthur, Switzerland), hereafter referred to as *Vicia*, *Chenopodium* and *Beta*. Seeds of *Vicia* and *Beta* were purchased, while the *Chenopodium* seeds were collected in the field in Zurich, Switzerland in autumn 2019. Within each host plant treatment, plants had the same age (*Vicia* 7 days, *Beta* and *Chenopodium* 27 days) and were chosen for most similar height and habitus. All plants are important summer hosts of *A*. *fabae fabae*, the nominal subspecies of *Aphis fabae* and a notorious agricultural pest (Blackman & Eastop, 2000).

We chose four aphid lines from our laboratory collection showing a broad spectrum of resistance to different lines of the parasitoid *L*. *fabarum*, as seen in previous studies (Cayetano & Vorburger, 2013a, 2013b, 2015). The four lines originate from a single clone of *A*. *fabae fabae* (clone ID: 407), which was collected in Switzerland in 2006 from *Chenopodium*. One of these lines was free of any facultative symbionts (407), and three lines were uniquely infected with one of three genetically different strains of *H*. *defensa*: H15, H76 and H402. To obtain these lines, the *H*.* defensa*‐free aphid clone 407 had been infected by microinjection of haemolymph from *H*.* defensa*‐carrying aphid clones (Cayetano & Vorburger, 2015), resulting in stable, heritable infections. The infected lines are referred to as 407^H15^, 407^H76^ and 407^H402^ and have been maintained parthenogenetically since the infection. Their identity was reconfirmed immediately before the experiment by microsatellite genotyping of the aphid (Coeur d’acier et al., 2004) and sequencing of the *H*. *defensa* gene murE (Degnan & Moran, 2008b). Using a single aphid clone should exclude genetic variation beyond the endosymbiont strain in the aphid lines. We included the *H*.* defensa*‐free aphid line in order to relate levels of *H*.* defensa*‐conferred resistance to the aphid's basal resistance.

As parasitoids, we used three asexual, isofemale lines of *L*. *fabarum* (06–242, 07–64 and 09–369), the most frequent parasitoid species of *A*.* fabae* in Switzerland (Rothacher et al., 2016). The lines had been started from single asexual females collected between 2006 and 2009. Parasitoid wasps oviposit single eggs into aphids. After hatching, the wasp larva feeds on the aphid's body, eventually kills the aphid and pupates within the emptied aphid exoskeleton. At this stage, a parasitized aphid is clearly recognizable and referred to as a mummy.

### 2.2 Experimental set‐up

We combined three host plants (*Vicia*, *Chenopodium*, *Beta*) with four aphid lines (407, 407^H15^, 407^H76^ and 407^H402^) and three parasitoid lines (06–242, 07–64 and 09–369) in a full factorial design with 36 treatment combinations and eight replicates, thus 288 experimental units. We performed the experiment in eight randomized complete blocks containing one replicate of each treatment. Four blocks were processed on the same day over two consecutive days. An experimental unit consisted of one plant populated by one aphid line, exposed to one parasitoid line. The plants were grown in pots of 5 cm diameter and covered with a ventilated plastic cup. The experiment was conducted in a climate chamber at constant 22°C with a 16‐h photoperiod.

We split up the four aphid lines to the 288 experimental units and reared them on the respective plants for two generations prior to the experiment, with each new generation being transferred to a new set of plants. These two generations of prior rearing for each replicate helped to level out potential environmental effects carried over from the stock cultures and allowed for physiological adaptation to the different plant species. To initiate the experimental generation, we put four adult female aphids on a plant on day 1. We let them reproduce for up to two days in order to have similar numbers of nymphs on each plant, before removing the adults and counting the nymphs on day 3. On day 4, we added two female parasitoids to each plant and removed them again after six hours. We then waited until the aphid mummies (= parasitized aphids) were clearly visible on day 13 and counted the parasitized as well as the adult non‐parasitized aphids. As response variable, we used the number of mummies divided by the number of nymphs initially exposed to the wasps (parasitism rate). A considerable number of aphids died in the time between the counting of the aphid nymphs and the counting of the mummies. To ensure that this would not falsify our conclusions, we did a second analysis with parasitism rate calculated as the number of mummies divided by the number of aphids still present (alive or parasitized) on the plant when counting mummies.

We further measured the fresh weight of the mothers of the experimental aphid generation on a precision balance (MX5, Mettler Toledo, Greifensee, Switzerland) to assess potential effects of host plant and aphid line on aphid size. As a measure connected to parasitoid fitness, we determined the proportion of wasps hatching from the mummies we collected during the experiment (emergence rate). For this, we cut the whole plant after having counted the mummies and kept it in an air permeable bag until the adult wasps emerged from the mummies.

### 2.3 Analysis

All statistical analyses were done in RStudio v1.2.5001 (RStudio Team, 2020) with R v3.6.1 (R Core Team, 2019) and using the package ggplot2 v3.3.2 (Wickham, 2016) for producing figures. To analyse parasitism rates, we used a generalized linear model with a logit link function and quasibinomial errors to account for overdispersion. We performed a three‐way factorial analysis of deviance testing for the effects of aphid line, parasitoid line and host plant as well as the two‐ and three‐way interactions, and for the main effect of experimental block. We used the function *Anova* from the R package *car* v3.0.7 (Fox & Weisberg, 2019) with *F* tests as recommended by Crawley (2014) for quasilikelihood fits. We treated experimental block as fixed since quasibinomial errors are not implemented for generalized mixed models in R. For consistency, we treated block as a fixed effect in all further analyses. While the initial number of nymphs exposed to the wasps differed between plants (*Beta* 15.6 ± 7.2 SD, *Chenopodium* 13.8 ± 6.0, *Vicia*: 17.3 ± 6.7), we did not include this value in the final model, since it had no significant effect on parasitism rates when aphid line and host plant were included (Table S1), suggesting that parasitoids were host limited (and not time limited) in our assays. We did the analysis once for the full data set (all aphid lines) and once with the data set restricted to the *H*.* defensa*‐infected aphid lines. Only in the latter case does the aphid line × parasitoid line interaction strictly reflect the G × G interactions between symbionts and parasitoids. Certain treatment combinations resulted in zero mummies in all replicates, and thus a group variance of zero, which led to problems with model convergence. To avoid this, we edited our data such that we manually added one mummy to one replicate of each ‘zero parasitism’ treatment combination. This minor intervention should have reduced treatment differences and hence made comparisons more conservative.

The parasitoid emergence rate was analysed with generalized linear models with logit link functions and binomial errors. Since we could analyse only samples where at least one mummy had formed, we performed separate analyses on data subsets from the aphid lines 407 and 407^H15^, where we had enough replicates, and tested only for the main effects of parasitoid line, host plant and block. Likewise, we tested for the main effects of aphid line, parasitoid line and block on a subset of the data including mummies collected from *Vicia* plants only. We calculated pairwise differences (Tukey HSD) between categories of significant predictors using the package *multcomp* v1.4.10 (Hothorn et al., 2008).

A model with quasibinomial errors as described above was also applied to analyse the proportion of aphids surviving until the end of the experiment among the non‐parasitized aphids (initially exposed aphids minus parasitized aphids). The log‐transformed aphid body weight measures were analysed using a linear model and an analysis of variance. Here, we tested for the effects of aphid line, host plant and the aphid × host plant interaction while accounting for the experimental block. We calculated pairwise differences (Tukey HSD) between aphid lines conditional on host plant using the R package *lsmeans* v2.30.0 (Lenth, 2016).

## 3 RESULTS

### 3.1 Parasitism rate and parasitoid emergence

**FIGURE 1:**
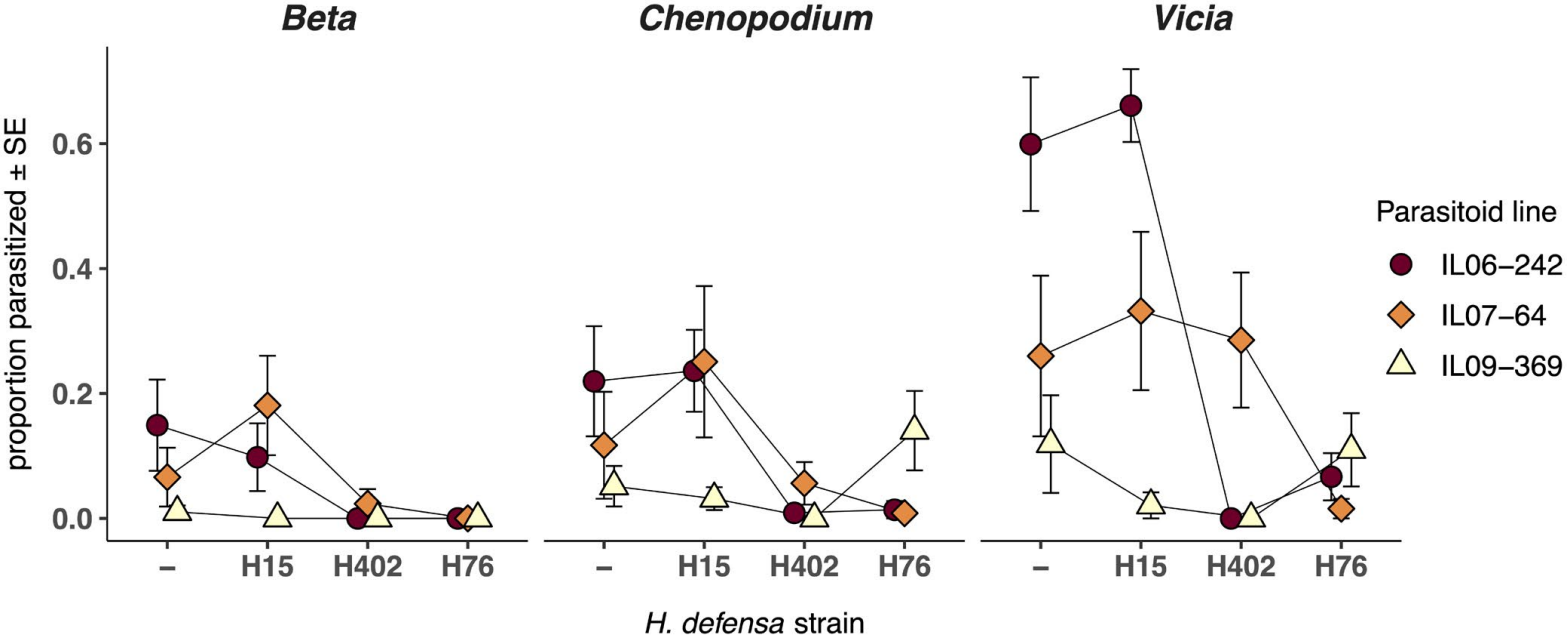
Parasitism rates calculated as number of mummies divided by number of exposed aphids. We used four lines of a single aphid clone: a line without *H*. *defensa* (407), and three infected with a different *H*. *defensa* strain each (lines 407^H15^, 407^H402^ and 407^H76^). The bars indicate standard errors

There were highly significant main effects of aphid line, parasitoid line and host plant on parasitism rates, as well as a significant block effect, both in the complete data set and in the data set restricted to *H*.* defensa*‐infected aphid lines (Table 1). The *H*. *defensa*‐free aphids and aphids carrying H15 were on average more susceptible to parasitoids than the aphids with the other two strains of *H*. *defensa*, and the observed rates of parasitism were mostly higher on *Vicia* than on the other two plants (Figure 1). The main effect of parasitoid line largely reflects the low parasitism success of line 09–369, even on aphids without *H*. *defensa*. In both analyses, parasitism rates were also strongly dependent on the specific combinations of host and parasitoid lines; i.e., there was a highly significant aphid line × parasitoid line interaction, which is uniquely determined by the G × G interaction between *L*. *fabarum* and *H*.* defensa* in the restricted data set (Table 1). There were no significant effects of the other interaction terms in the full data set, while the parasitoid × plant interaction was marginally significant in the restricted data set. Most notably with regard to the study question, the three‐way interaction between aphid line, parasitoid line and host plant was non‐significant in both analyses (Table 1). Hence, we observed no evidence for a G × G × E interaction on parasitism rates, indicating that the genetic specificity of the interaction between *H*. *defensa* and *L*. *fabarum* is not significantly altered by the different host plant environments. These conclusions remain unchanged when parasitism rates are calculated as the proportion of parasitized aphids among parasitized and surviving adult aphids at the end of the experiment, i.e. excluding aphids that died of reasons other than parasitism (Table S2).

**TABLE 1:** Analysis of deviance table for the proportion of aphids parasitized (parasitism rate)

Effect	(a) All aphid lines				(b) *H*.* defensa*‐infected lines			
	df	Sum Sq	*F*	*p*	df	Sum Sq	*F*	*p*
Block	7	112.86	4.649	<0.001	7	99.57	5.816	<0.001
Aphid	3	308.42	29.644	<0.001	2	224.31	45.858	<0.001
Parasitoid	2	213.55	30.790	<0.001	2	110.82	22.656	<0.001
Plant	2	287.40	41.437	<0.001	2	125.69	25.696	<0.001
Aphid × parasitoid	6	208.15	10.003	<0.001	4	190.38	19.461	<0.001
Aphid × plant	6	10.78	0.518	0.794	4	8.77	0.8961	0.467
Parasitoid × plant	4	21.84	1.574	0.182	4	24.54	2.509	0.044
Aphid × parasitoid × plant	12	22.51	0.541	0.887	8	15.25	0.780	0.621
Residual	245	849.65			182	445.11		

Note

A generalized linear model with logit link and quasibinomial fit was applied. (a): results using the full data set including all four aphid lines (288 samples), the dispersion parameter is 3.468. (b): results for including only the three *H*.* defensa*‐infected aphid lines (216 samples), the dispersion parameter is 2.446.

**FIGURE 2:**
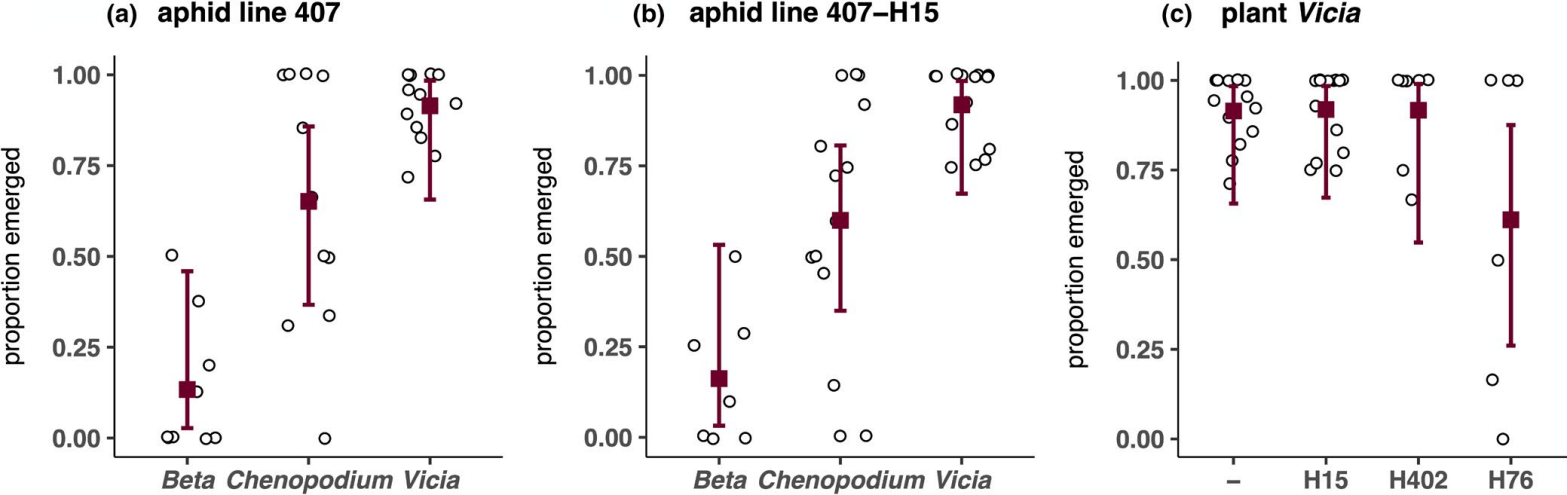
Proportion of parasitoids that emerged from the collected mummies, averaged over all parasitoid lines, for three data subsets: aphid lines 407 and 407^H15^, and plant *Vicia*. Single datapoints are shown as white dots, the dark squares show the mean per host plant (a, b) or *H*. *defensa* strain associated with the aphid clone 407 (c), respectively. The bars indicate 95% confidence intervals

Parasitoid emergence from parasitized aphids differed among treatments. Considering data from aphid lines 407 and 407^H15^ separately, parasitoid emergence was in both cases significantly influenced by the host plant, and to a lower extent also by the parasitoid line in aphid line 407^H15^ (Figure 2a, b, Table 2, Table S3). Mean parasitoid emergence for 407 and 407^H15^, respectively, was highest on *Vicia* (91% and 92%), intermediate on *Chenopodium* (65% and 60%) and lowest on *Beta* (13% and 16%). Considering data from *Vicia* plants only, there was a significant main effect of block and aphid line on emergence rate, and no effect of parasitoid line (Table 2). The effect of aphid line reflects a lower emergence from mummies of the aphid line 407^H76^ (61% emerged on *Vicia*) compared to all other aphid lines (91–92% emerged on *Vicia*) (Figure 2c, Table S3).

**TABLE 2:** Analysis of deviance table for the parasitoid emergence rate. Generalized linear models with logit link and binomial errors were applied on three data subsets covering sufficient replicates

Data subset	Effect	df	LR χ^2^	*p*
Aphid line 407	Block	7	13.84	0.054
	Parasitoid	2	1.76	0.416
	Plant	2	70.95	<0.001
Aphid line 407^H15^	Block	7	8.76	0.270
	Parasitoid	2	8.40	0.015
	Plant	2	62.92	<0.001
Plant *Vicia*	Block	7	24.96	<0.001
	Aphid	3	13.25	0.004
	Parasitoid	2	1.36	0.508

### 3.2 Aphid survival and body weight

From all initially exposed aphid nymphs, one part got mummified, one part survived, and one part died before the end of the experiment for reasons other than visible mummification (Figure S1). Among the non‐parasitized aphids, the proportion of surviving aphids varied significantly, explained by a significant main effect of host plant and experimental block (Table 3). Averaged over all parasitoid and aphid lines, 59% of all aphids survived on *Vicia*, 57% on *Chenopodium* and 36% on *Beta*.

**TABLE 3:** Analysis of deviance table for the proportion of aphids surviving until the end of the experiment among the non‐parasitized aphids

Effect	df	Sum Sq	*F*	*p*
Block	7	88.45	3.257	0.003
Aphid	3	9.59	0.824	0.481
Parasitoid	2	5.04	0.649	0.523
Plant	2	426.03	54.901	<0.001
Aphid × parasitoid	6	13.23	0.568	0.756
Aphid × plant	6	12.48	0.536	0.781
Parasitoid × plant	4	37.11	2.391	0.051
Aphid × parasitoid × plant	12	56.83	1.221	0.269
Residual	244	946.71		

Note

A generalized linear model with logit link and quasibinomial errors was applied; the dispersion parameter was 3.880.

**FIGURE 3:**
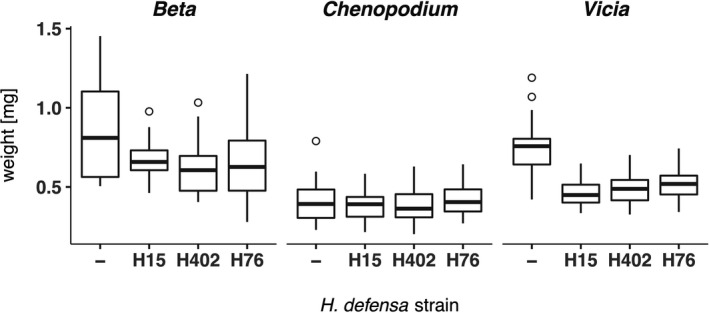
Adult weight of the mothers of the experimental aphid generation, in milligrams. Aphids on *Beta* had the highest weight on average, aphids on *Chenopodium* the lowest. Boxplot hinges correspond to the 1st and 3rd quartiles, the whiskers extend to a length of 1.5 times the inter‐quartile range. *x*‐axis: endosymbiotic *H*. *defensa* strain associated with the aphid clone 407

The aphids also varied in body weight, with significant main effects of aphid line (*F*_3,259_ = 15.37, *p* < 0.001), host plant (*F*_2,259_ = 109.97, *p* < 0.001) and experimental block (*F*_7,259_ = 4.50, *p* < 0.001), as well as a significant aphid line × host plant interaction (*F*_6,259_ = 4.08, *p* < 0.001). The effect of aphid line and the interaction effect manifest in lower weight of the *H*.* defensa*‐infected aphid lines compared to the *H*. *defensa*‐free aphid line on *Vicia* and *Beta*, but not on *Chenopodium* (Figure 3, Table S4). On average, aphid weight was highest on *Beta* (0.704 mg ± 0.233 mg SD), followed by *Vicia* (0.555 mg ±0.161 mg SD) and lowest on *Chenopodium* (0.400 mg ± 0.110 mg SD).

## 4 DISCUSSION

Genotype‐by‐genotype interactions between the parasitoid *L*. *fabarum* and the aphid‐protective endosymbiont *H*. *defensa* have been observed in multiple laboratory experiments (Cayetano and Vorburger, 2015; Schmid et al., 2012) and are therefore assumed to be an important driver of the coevolutionary dynamics in this host–parasitoid system (Hafer & Vorburger, 2019; Hafer‐Hahmann & Vorburger, 2020; Kwiatkowski et al., 2012). Here, we investigated such G × G interactions on different host plants, a key environmental variable for aphids and their parasitoids in natural populations. We observed the expected G × G interactions on all host plants, but also a strong main effect of the host plants on overall parasitism rates. In contrast, we saw no significant G × G × E interaction, suggesting that environmental heterogeneity generated by the availability of different host plants in the field does not reduce the hierarchy of G × G interactions between *L*.* fabarum* and *H*.* defensa*. Taken together, these findings imply that host plant variation across space and time can indeed create a mosaic of varying selection strength (Thompson, 2005), but without changing the specificity of reciprocal selection between symbiont‐protected hosts and parasitoids. Coevolutionary dynamics may proceed at different pace in different host plant environments, but the environmental variation is unlikely to change the direction of selection (Wolinska & King, 2009). Our findings thus resemble those of Cayetano and Vorburger (2013b), who assessed G × G interactions in the same system, but at different ambient temperatures. They found a clear effect of temperature on overall parasitism rates, but no evidence for a G × G × E interaction. While investigating two completely different aspects of the environment, the two studies reinforce each other in that the G × G interactions between *H*. *defensa* and *L*. *fabarum* are very robust to environmental perturbation.

While parasitism rates were affected by the plant they were measured on, the protective effect conferred by the different *H*. *defensa* strains remained similar on all plants (no aphid × plant interaction; Table 1, Figure 1). This may not be surprising considering the mechanistic basis underlying *H*.* defensa*‐conferred resistance, which is related to the presence of a bacteriophage, called APSE, within the bacterial genome (Oliver et al., 2009). Different APSE types carry specific toxin cassettes, which encode for different putative toxins, likely responsible for variation in the protective phenotype among *H*. *defensa* strains (Degnan & Moran, 2008a; Moran, Degnan, et al., 2005; Oliver et al., 2009; Oliver & Higashi, 2019). The three strains used here also represent clearly distinct genotypes (Kaech et al., 2021). The phage toxins are assumed to kill susceptible parasitoids at an early stage, that is as eggs or early larvae, but they may also have later‐acting effects when parasitoids manage to complete development despite the presence of *H*. *defensa*, such as reduced adult weight or delayed emergence of parasitoids (Dennis et al., 2017; Schmid et al., 2012). Such a late‐acting detrimental effect may explain the low parasitoid emergence rate from mummies of the aphid line 407^H76^ (Figure 2, Table S2), which was already observed in an earlier experiment (Schmid et al., 2012).

Infection with a toxin‐producing symbiont can also be associated with costs to the host, and indeed, the frequency of *H*.* defensa*‐carrying aphids tends to decline within aphid populations that are not under selection by parasitoids (Dykstra et al., 2014; Hafer‐Hahmann & Vorburger, 2020; Oliver et al., 2008). Here, we measured adult weight as a rough proxy for aphid performance and found that *H*.* defensa*‐free aphids were clearly larger than *H*.* defensa*‐carrying ones on *Vicia* and *Beta*, but not on *Chenopodium*, where aphids were generally smaller. This was reflected in a significant aphid line × host plant interaction, indicating that the cost of infection with *H*. *defensa* may depend on the host plant an aphid is feeding on. Host plant‐dependent costs of symbiont‐conferred resistance would also have the potential to affect host–parasitoid coevolution, in that the net cost or benefit of possessing a resistance‐conferring symbiont would vary across a geographic mosaic of host plant availability.

That the stability of the observed G × G interactions between *H*. *defensa* and *L*. *fabarum* is not simply a result of weak environmental differences is indicated by more than the aphid body weight varying between host plants. Despite the low parasitism rate on *Beta*, aphid mortality was clearly elevated there compared to *Chenopodium* or *Vicia* (Table 3, Figure S1). Moreover, differences in the reproductive success of parasitoids due to lower parasitism rates on *Beta* and *Chenopodium* were amplified further by variation in emergence rates, which were also lowest on *Beta* and intermediate on *Chenopodium* (Figure 2a, b, Table S2). In summary, *Vicia* was the most favourable host plant for aphids as well as parasitoids, while *Beta* represented a comparatively adverse environment for both antagonists. We could thus expect that the strength of reciprocal selection between hosts and parasitoids is higher on relatively benign host plants such as *Vicia*.

How do differences in host plant quality for both antagonists come about mechanistically? For the parasitoids, differences in parasitism success may be related to variation in plant structure affecting how efficiently aphids are attacked (Grevstad & Klepetka, 1992; Kareiva & Sahakian, 1990), or to variation in host quality (Pan et al., 2020). The entire parasitoid development takes places within the aphid's body; hence, the more vital and well‐fed the aphids are, and the more resources may be available for the parasitoid. A resource deficit compared with *Vicia*‐feeding aphids is a possible explanation for the low parasitism success on low‐weight aphids from *Chenopodium*, but another explanation must apply to the frequent parasitoid failure on aphids from *Beta*, which were even heavier on average than the aphids from *Vicia*. *Beta* leaves may contain high concentrations of oxalates (Baker & Eden, 1954), which can have negative effects on aphids (Massonié, 1980) and could thus be responsible for the lower survival in our experiment, but of course many other reasons are conceivable. If the lower vitality of *Beta*‐feeding aphids came from the uptake of some toxic plant compound, this may also have hampered the parasitoids’ development (Turlings & Benrey, 1998). The low aphid survival on *Beta* in our experiment was surprising, though, since *A*. *fabae* is known as a severe pest in sugar beet cultures (Blackman & Eastop, 2000), and we indeed regularly observe heavy infestations of sugar beet during fieldwork. However, the species *Beta vulgaris* unites a multitude of crop varieties which may differ in their quality as host plants for *A*. *fabae*. The green chard variety we used in this experiment may be a less favourable host plant than the commonly grown varieties of sugar beet.

Whatever the precise reasons, *Vicia*, *Chenopodium* and *Beta* represented very different environments for the aphids and the parasitoids, and still, the genetic interactions tested within remained virtually unaffected. This is not self‐evident, as several studies examining host–parasite interactions under different environmental conditions have reported significant G × G × E effects (Bryner & Rigling, 2011; Piculell et al., 2008; Sadd, 2011; Tétard‐Jones et al., 2007; Wendling et al., 2017; Zouache et al., 2014). Explicit reports of the lack of a G × G × E interaction in host–parasite systems are scarce to our knowledge (but see Cisarovsky et al. 2012 and Cayetano and Vorburger 2013b). To some extent, this may reflect a publication bias against reporting negative results (Csada et al., 1996). We argue that the absence of a significant G × G × E interaction is relevant here because it corroborates the importance we may attribute to the G × G effects. The more robust G × G interactions are to environmental variability, the more pervasive they will be in natural populations, and thus, the more likely they explain fundamental evolutionary phenomena, such as the maintenance of genotypic diversity (Hafer & Vorburger, 2019; Judson, 1995). G × G interactions between *H*. *defensa* and *L*. *fabarum* have been shown to persist over a range of average temperatures and, as we newly report here, aphid host plants. This consolidates the role of the defensive symbiont *H*. *defensa* as a key mediator of coevolution between aphids and parasitoids, also in a heterogeneous environment.

## CONFLICT OF INTEREST

The authors have no conflict of interest to declare.

## AUTHOR CONTRIBUTIONS

EG and CV designed the study. EG conducted the experiment. Both authors contributed to data analysis and interpretation. EG wrote the first draft of the manuscript, which was edited and revised by both authors.

### PEER REVIEW

The peer review history for this article is available at https://publons.com/publon/10.1111/jeb.13953.

## Supporting information

Fig S1

Table S1‐S4

## Data Availability

The data set generated in this study is available at Dryad Digital Repository: https://doi.org/10.5061/dryad.2ngf1vhpd
