# Supplementary figures and images for: Strong genotype‐by‐genotype interactions between aphid‐defensive symbionts and parasitoids persist across different biotic environments
Source: J Evol Biol. 2021 Nov 2;34(12):1944–53. doi: 10.1111/jeb.13953 (PMC9298302; doi:10.1111/jeb.13953)

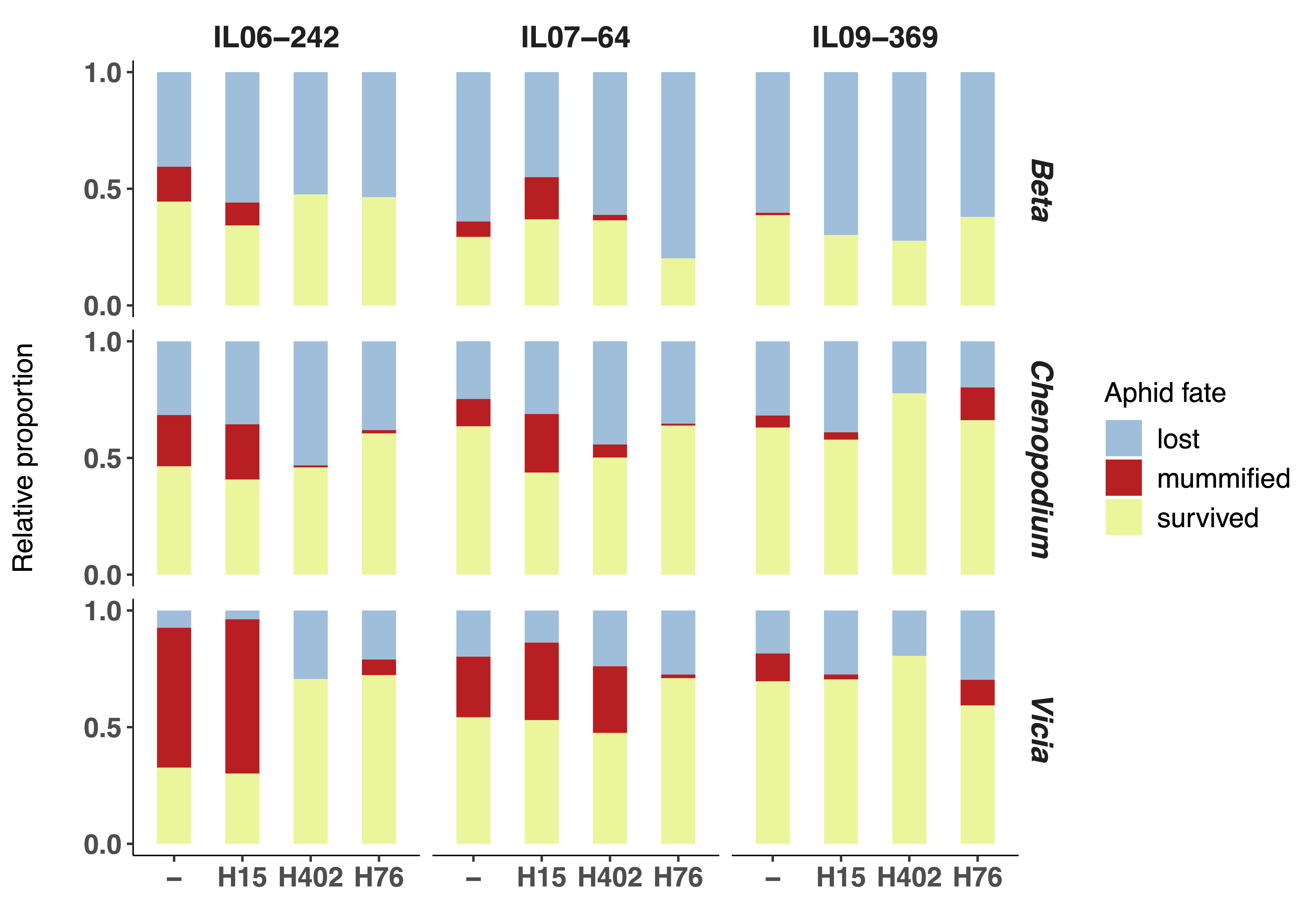

Supplement: Supplementary file 1 — Fig S1 [file JEB-34-1944-s002.jpg]
