# Supplementary material for: Strong genotype‐by‐genotype interactions between aphid‐defensive symbionts and parasitoids persist across different biotic environments
Source: J Evol Biol. 2021 Nov 2;34(12):1944–53. doi: 10.1111/jeb.13953 (PMC9298302; doi:10.1111/jeb.13953)
Supplement: Supplementary file 2 — Table S1‐S4 [file JEB-34-1944-s001.docx]

**Supplementary Materials: Tables**

**Table S1.** Analysis of deviance table for the proportion of aphids parasitized (parasitism rate), applying a generalized linear model with logit link and quasibinomial fit. In contrast to the analysis presented in Table 1, we included the number of nymphs exposed to the parasitoids (“nymphs”) here. Since “nymphs” is not significantly contributing to the model as long as “plant” and “aphid” are included, we did not keep the variable in the final model presented in Table 1. Column A: results using the full dataset including all four aphid lines (288 samples), the dispersion parameter is 3.666. Column B: results for including only the three *H. defensa*-infected aphid lines (216 samples), the dispersion parameter is 2.436.

|  | **A. All aphid lines** | | | |  | | **B. *H. defensa*-infected lines** | | | |
| --- | --- | --- | --- | --- | --- | --- | --- | --- | --- | --- |
| Effect | df | Sum Sq | F | P | |  | df | Sum Sq | F | P |
| Block | 7 | 109.31 | 4.260 | <0.001 | |  | 7 | 101.86 | 5.974 | <0.001 |
| Nymphs | 1 | 8.09 | 2.207 | 0.139 | |  | 1 | 2.29 | 0.941 | 0.333 |
| Aphid | 3 | 275.04 | 25.011 | <0.001 | |  | 2 | 225.74 | 46.339 | <0.001 |
| Parasitoid | 2 | 218.64 | 29.823 | <0.001 | |  | 2 | 110.95 | 22.774 | <0.001 |
| Plant | 2 | 270.08 | 36.840 | <0.001 | |  | 2 | 128.56 | 26.390 | <0.001 |
| Aphid × parasitoid | 6 | 208.32 | 9.472 | <0.001 | |  | 4 | 190.24 | 19.526 | <0.001 |
| Aphid × plant | 6 | 9.60 | 0.437 | 0.854 | |  | 4 | 9.32 | 0.956 | 0.433 |
| Parasitoid × plant | 4 | 22.49 | 1.534 | 0.193 | |  | 4 | 23.47 | 2.409 | 0.051 |
| Aphid × parasitoid × plant | 12 | 24.21 | 0.551 | 0.880 | |  | 8 | 14.98 | 0.769 | 0.631 |
| Residual | 244 | 894.41 |  |  | |  | 181 | 440.87 |  |  |

**Table S2.** Analysis of deviance table for the proportion of aphids parasitized (parasitism rate). Here, the proportion is calculated as mummies / (mummies + surviving_aphids), while for Table 1 the proportion is calculated as mummies / exposed_aphids. A generalized linear model with logit link and quasibinomial fit was applied. Column A shows the results for analyzing the full dataset including all four aphid lines (279 samples, 9 samples were excluded compared to the analysis in Table 1 since (mummies + surviving_aphids) = 0); the dispersion parameter was 2.931. Column B shows the results for analyzing only the *H. defensa*-infected aphid lines (208 samples), the dispersion parameter was 2.136.

|  | **A. All aphid lines** | | | |  | | **B. *H. defensa*-infected lines** | | | |
| --- | --- | --- | --- | --- | --- | --- | --- | --- | --- | --- |
| Effect | df | Sum Sq | F | P | |  | df | Sum Sq | F | P |
| Block | 7 | 156.40 | 7.623 | <0.001 | |  | 7 | 136.38 | 9.119 | <0.001 |
| Aphid | 3 | 270.96 | 30.813 | <0.001 | |  | 2 | 204.37 | 47.828 | <0.001 |
| Parasitoid | 2 | 177.13 | 30.214 | <0.001 | |  | 2 | 86.72 | 20.296 | <0.001 |
| Plant | 2 | 99.99 | 17.056 | <0.001 | |  | 2 | 33.13 | 7.754 | <0.001 |
| Aphid × parasitoid | 6 | 199.01 | 11.315 | <0.001 | |  | 4 | 183.24 | 21.442 | <0.001 |
| Aphid × plant | 6 | 18.25 | 1.038 | 0.401 | |  | 4 | 15.90 | 1.861 | 0.119 |
| Parasitoid × plant | 4 | 14.63 | 1.248 | 0.292 | |  | 4 | 17.19 | 2.011 | 0.095 |
| Aphid × parasitoid × plant | 12 | 21.50 | 0.611 | 0.832 | |  | 8 | 16.36 | 0.957 | 0.471 |
| Residual | 236 | 691.77 |  |  | |  | 174 | 371.75 |  |  |

**Table S3.** Pairwise post hoc Tukey HSD of the parasitoid emergence rate between pairs of significant predictors as resulting from the analysis of deviance presented in Table 2. A generalized linear fit with logit link and binomial errors was used, the estimates are given at the logit (not response) scale.

| Data subset | Contrast | Estimate | SE | Z | P |
| --- | --- | --- | --- | --- | --- |
| Aphid line | Cheno – Beta | 1.540 | 0.714 | 2.158 | 0.078 |
| **407** | Vicia – Beta | 4.075 | 0.651 | 6.259 | <0.001 |
|  | Vicia – Cheno | 2.535 | 0.572 | 4.434 | <0.001 |
|  |  |  |  |  |  |
| Aphid line | Cheno – Beta | 2.337 | 0.609 | 3.838 | <0.001 |
| **407-H15** | Vicia – Beta | 4.148 | 0.662 | 6.263 | <0.001 |
|  | Vicia – Cheno | 1.811 | 0.438 | 4.132 | <0.001 |
|  |  |  |  |  |  |
|  | IL07-64 – IL06-242 | 0.848 | 0.434 | 1.955 | 0.099 |
|  | IL09-369 – IL06-242 | 18.493 | 2751.987 | 0.007 | 1.000 |
|  | IL09-369 – IL07-64 | 17.644 | 2751.987 | 0.006 | 1.000 |
|  |  |  |  |  |  |
| Plant | 407H15 – 407 | 0.192 | 0.411 | 0.467 | 0.963 |
| ***Vicia*** | 407H402 – 407 | 0.632 | 0.746 | 0.847 | 0.818 |
|  | 407H76 – 407 | -2.555 | 0.761 | -3.358 | 0.004 |
|  | 407H402 – 407H15 | 0.440 | 0.724 | 0.607 | 0.923 |
|  | 407H76 – 407H15 | -2.748 | 0.821 | -3.348 | 0.004 |
|  | 407H76 – 407H402 | -3.187 | 1.054 | -3.023 | 0.012 |

**Table S4.** Pairwise post hoc Tukey HSD of aphid fresh weight between aphid lines, per host plant. We used the command lsmeans(wmodel, pairwise ~ a_clone | plant) from the R package *lsmeans* v﻿2.30.0. We used a linear fit on log-transformed weight measures, thus the estimates are given on the log (not response) scale.

| Plant | Contrast | Estimate | SE | df | t ratio | P |
| --- | --- | --- | --- | --- | --- | --- |
| *Beta* | 407 - 407H15 | 0.194 | 0.077 | 259 | 2.537 | 0.057 |
|  | 407 - 407H402 | 0.257 | 0.077 | 259 | 3.358 | 0.005 |
|  | 407 - 407H76 | 0.258 | 0.076 | 259 | 3.406 | 0.004 |
|  | 407H15 - 407H402 | 0.063 | 0.078 | 259 | 0.811 | 0.849 |
|  | 407H15 - 407H76 | 0.064 | 0.077 | 259 | 0.831 | 0.840 |
|  | 407H402 - 407H76 | 0.001 | 0.077 | 259 | 0.010 | 1.000 |
|  |  |  |  |  |  |  |
| *Chenopodium* | 407 - 407H15 | 0.053 | 0.072 | 259 | 0.732 | 0.884 |
|  | 407 - 407H402 | 0.073 | 0.072 | 259 | 1.002 | 0.748 |
|  | 407 - 407H76 | -0.017 | 0.072 | 259 | -0.237 | 0.995 |
|  | 407H15 - 407H402 | 0.020 | 0.072 | 259 | 0.270 | 0.993 |
|  | 407H15 - 407H76 | -0.070 | 0.072 | 259 | -0.969 | 0.767 |
|  | 407H402 - 407H76 | -0.090 | 0.072 | 259 | -1.239 | 0.603 |
|  |  |  |  |  |  |  |
| *Vicia* | 407 - 407H15 | 0.474 | 0.072 | 259 | 6.544 | <0.001 |
|  | 407 - 407H402 | 0.423 | 0.072 | 259 | 5.840 | <0.001 |
|  | 407 - 407H76 | 0.369 | 0.072 | 259 | 5.101 | <0.001 |
|  | 407H15 - 407H402 | -0.051 | 0.072 | 259 | -0.704 | 0.896 |
|  | 407H15 - 407H76 | -0.104 | 0.072 | 259 | -1.443 | 0.473 |
|  | 407H402 - 407H76 | 0.054 | 0.072 | 259 | 0.740 | 0.881 |
|  |  |  |  |  |  |  |

**Supplementary Materials: Figure Caption**

**Figure S1**. Proportion of surviving aphids (green, bottom color), parasitized aphids (red, middle color) and aphids dying for reasons other than visible mummification (blue, top color), out of all initially exposed aphids. Rows represent host plants; main columns represent parasitoid lines (IL06-242, IL07-64 and IL09-369); and single bars correspond to the endosymbiotic *H. defensa* strain associated with the aphid clone 407 (none, H15, H402 or H76).
